# A bibliometric analysis of Mpox research based on Web of Science platform

**DOI:** 10.1097/MD.0000000000043329

**Published:** 2025-07-11

**Authors:** Qunjiao Yan, Lei Wang

**Affiliations:** a Academy of Military Sciences, Academy of Military Medical Sciences, Beijing, China.

**Keywords:** bibliometrics, knowledge map, Mpox, public health, visual analysis

## Abstract

Mpox is still a public health emergency of international concern. A bibliometric and knowledge mapping analysis were performed to systematically examine the Mpox research landscape. Mpox-related literature was retrieved from the core collection of the Web of Science database. This study conducts a statistical analysis of related publications, examining the distribution patterns across publication years, category, journals, institutions, and authors. And CiteSpace, VOSviewer, and data-information-knowledge-wisdom were used to extract information about countries/regions, institutions, authors, category, and keywords to identify and analyze the research hotspots and trends in this field. A total of 3401 Mpox-related articles were retrieved, and since 2022, the volume of literature has increased sharply. The United States was the main publishing country, with the highest number of publications and citations. Infectious diseases and virology were the main research disciplines. Two major cooperation clusters had emerged, centered on China and the U.S., with the U.S.-led research cluster showing a multi-theme parallel advancement in thematic research. The United States Centers for Disease Control and Prevention was the most prolific institution, and its affiliated researcher Damon IK is the most productive author. *Viruses-Basel* has the highest number of publications. The epidemiology and public health regulation, genetic evolution, and transmission mechanism, pathogenesis and host immunity, drug research, and vaccine effectiveness were the main research topics. The study of Mpox transmission mechanism combined with ecology and artificial intelligence-based diagnostic methods are emerging research directions. Bibliometric analysis of Mpox studies enables researchers to efficiently identify research landscapes and emerging trends, providing critical references for countries to effectively allocate research priorities and identify collaborative partners, offering valuable insights for formulating epidemic containment strategies.

## 1. Introduction

Mpox virus is a zoonotic double-stranded DNA virus that belongs to the genus *Orthopoxvirus*, demonstrating both primary transmission between animal and human populations, as well as secondary human-to-human transmission.^[1,2]^ As early as 1953, Reagan et al^[3]^ focused on the electron microscopic study of chickenpox virus in monkey serum, and Mpox was officially named in 1958 when the virus was first identified in infected macaques in Copenhagen, Denmark. Mpox virus, initially named monkeypox, showed a broad propensity to infect mammals.^[4]^ In 1970, the first case of monkeypox infection in human was diagnosed in the Democratic Republic of Congo. The geographical location of the outbreak indicates that Mpox originated from western and central African countries. However, with increasing international trade and business travel, confirmed cases have been reported in several countries since 2016. Mpox outbreaks have increased in scope and frequency since 2022.^[5]^ The World Health Organization (WHO) declared the Mpox outbreak a global public health emergency on July 23, 2022.^[6]^ In the same year, “monkeypox” was renamed “Mpox,” the Congo Basin clade was renamed Clade I, the West African clade was renamed Clade II, and the 2 subclades of Clade II were named ІІa and ІІb.^[7]^ In August 14, 2024, WHO director general declared Mpox outbreak a public health emergency of international concern again. Mpox is gaining heightened attention in both social and public health spheres.

Since Mpox was reported, many related literatures have been published. Mohapatra RK et al emphasized the transmission dynamics, zoonosis potential, complication and mitigation strategies for Mpox infection, and they concluded with a recommendation for a “One Health” approach to enhance the management, control, and prevention of the disease.^[8]^ Zeeshan HF et al^[9]^ provided an essential insight into the research response to scientific trends of Mpox based Scopus database. A limited number of studies have employed bibliometric methods to analyze Mpox research up to 2022.^[10,11]^ However, there is currently a paucity of research focusing up-to-date on Mpox.

Bibliometric analysis is a research methodology that systematically collects quantifiable, reproducible, and objective data to investigate scientific activities and developmental trends within specific research domains. This statistical technique has gained significant recognition due to its distinctive advantages and broad applicability across multiple disciplines, where it has been extensively employed to identify diverse research patterns and scientific regularities.^[12–14]^

In the current study, we aimed to use bibliometric analysis to outline the historical progress, current research status, and future development trends in Mpox field and analyzed the category, journals, countries, institutions, authors, and years of monkeypox related articles. Thus, in this study, we reviewed the literature on Mpox-related based on the Web of Science (WoS). So, what about the research situation of Mpox? To identify the most relevant topics and trends in Mpox research, we addressed the following research questions: What is the distribution of Mpox studies by year of publication? What’s the disciplinary distribution within the Mpox research field and the change pattern? Which are the most productive authors, institutions, and countries? What is the pattern of international collaboration and national research preferences in the Mpox research field? What are the characteristics and developmental trends in Mpox research? We hope this study can provide valuable reference information for scholars aiming to conduct further Mpox related research and as a source for those seeking information about Mpox as well as add a new reference for future human Mpox prevention.

## 2. Material and methods

### 2.1. Data sources

Data for this study were retrieved from the core collection of WoS database, using a subject search with the following search formula: ((((((TS = (Mpox)) OR TS = (MPXV)) OR TS = (Monkeypox)) OR TS = (Monkeypoxvirus*)) OR TS=(“Monkeypox virus*”)) OR TS=(“Monkey pox virus*”)) OR TS=(“MPX virus*”). The type of documentation is limited to article, letter, review, and meeting abstract, and proceeding paper, a total of 3401 papers were retrieved on March 22, 2024.

### 2.2. Methods

VOSviewer,^[15]^ CiteSpace,^[16]^ data-information-knowledge-wisdom^[17]^ were used for data cleaning, data analysis, and visualization of the retrieved documents. Analysis of generated graphs in this study was combined with manual literature reading.

The data used in this study were from the WoS and did not involve patients. Therefore, permission was not required from the ethics committee.

## 3. Results

### 3.1. Analysis of the annual number of publications on Mpox

**Figure 1.**
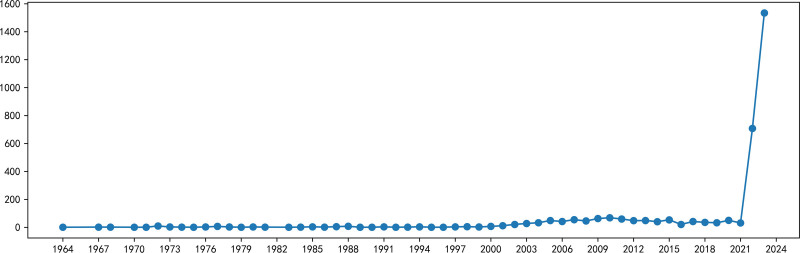
Annual trend in the number of published papers.

The annual number of publications on Mpox is shown in Figure 1. From 1953 to 2000, the number of publications on this topic was low, with an average annual number of 3.5.

As observed in Figure 1, the highest number of publications (10) before 2000 was reached in 1972. After 2000, the number of publications on Mpox has increased, especially after the first cases were reported outside Africa by the Centers for Disease Control and Prevention (CDC) in 2003.^[18]^ In 2017, a Mpox outbreak occurred in Nigeria, and on November 3, 2017, the WHO held an informal consultation on Mpox in Geneva, which focused on assessing the situation of the Mpox epidemic and state of relevant knowledge and identifying gaps in the fight against Mpox.^[19]^ This meeting reflected the growing international concern about Mpox. As we can see from Figure 1, the number of published papers was maintained at a high level around 2017. In 2022, the world experienced a major Mpox outbreak. Following a confirmed case of Mpox with a history of travel to Nigeria reported by the UK Health Security Agency on May 6, 2022, many countries reported confirmed cases, including the first imported cases reported in mainland China on September 16, 2022. According to the WHO, most cases were from America and Africa.^[20]^ The 2022 outbreak led to a rapid increase in papers related to Mpox, with over 700 documents published in 2022. Mpox continued to gain traction, with more than twice as many articles published in 2023 as in 2022. Overall, the number of Mpox-related publications began to increase after 2000. Since the global Mpox outbreak in 2022, there has been a surge in research papers on the topic.

### 3.2. Analysis of the disciplinary distribution of Mpox

Subject analysis results from the WoS database (Table 1) showed that infectious diseases and virology were the main research disciplines, accounting for 20.80% and 15.34% of all Mpox disciplines, respectively. Other areas that contributed more than 10% of publications included immunology (13.62%), public environmental occupational health (12.99%), microbiology (11.72%). Among Mpox disciplines published from 2008 to 2021, virology was the top-ranked discipline, and in 2022, the infectious disease was the top-ranked discipline, with microbiology, immunology, internal general medicine, and public environmental occupational health, ranking ahead of virology (Appendix Fig. S1, Supplemental Digital Content, https://links.lww.com/MD/P409). This shift in discipline ranking can be attributed mainly to the Mpox outbreak in 2022; therefore, the research tilted from basic to applied studies.

**Table 1:** Statistics of published papers on Mpox by subjects.

Number	Subject	Number of publications
1	Infectious Diseases	724
2	Virology	534
3	Immunology	474
4	Public Environmental Occupational Health	452
5	Microbiology	408
6	Medicine General Internal	294
7	Medicine Research Experimental	199
8	Pharmacology Pharmacy	172
9	Biochemistry Molecular Biology	161
10	Multidisciplinary Sciences	140
11	Tropical Medicine	122
12	Biotechnology Applied Microbiology	112
13	Health Care Sciences Services	74
14	Dermatology	72
15	Parasitology	68

### 3.3. Country analysis of the documents on Mpox

#### 3.3.1. Analysis of national cooperation networks

**Figure 2.**
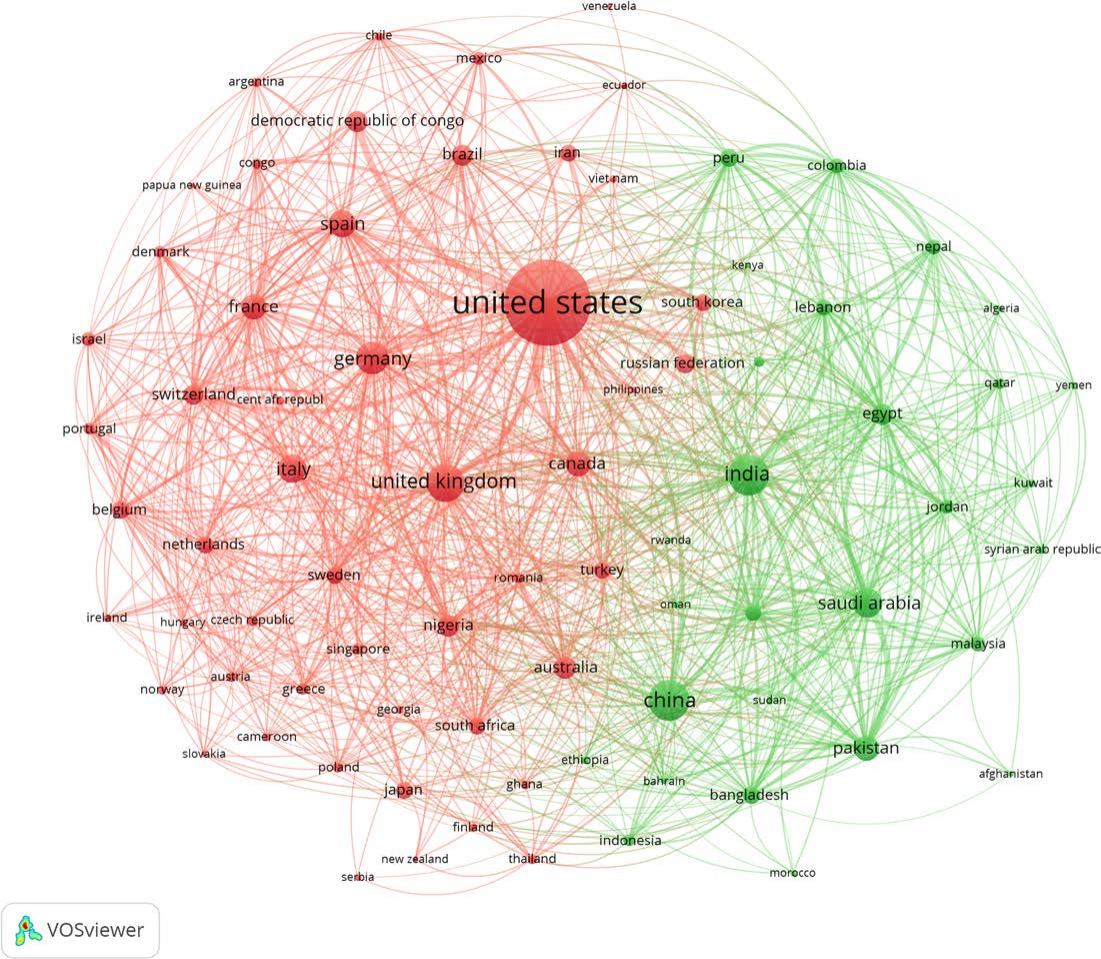
Map of national cooperation network on Mpox.

VoSviewer was used to draw the national cooperation map of Mpox research papers. As shown in Figure 2, the size of the nodes represents the publication frequency, and the thickness of the connecting lines represents the intensity of cooperation. The United States had the highest number of publications, and 2 main country clusters emerged, 1 centered on China and the other on the United States. The China-centered country cluster comprised of India, Saudi Arabia, Pakistan, Egypt, Peru, Bangladesh, Lebanon, United Arab Emirates, Malaysia, and Colombia, while the United States-centered country cluster included the United Kingdom, Germany, Italy, Spain, Canada, France, Australia, Brazil, Nigeria, and Democratic Republic of Congo. As observed in Figure 2, the U.S.-centered cluster demonstrates stronger research capabilities. Some collaboration also exists between the 2 country clusters indicating there are channels for sharing information, knowledge, resources, and technology between countries, which are conducive to the prevention and detection of and response toward Mpox disease worldwide.

#### 3.3.2. Topic analysis of national concerns

**Figure 3.**
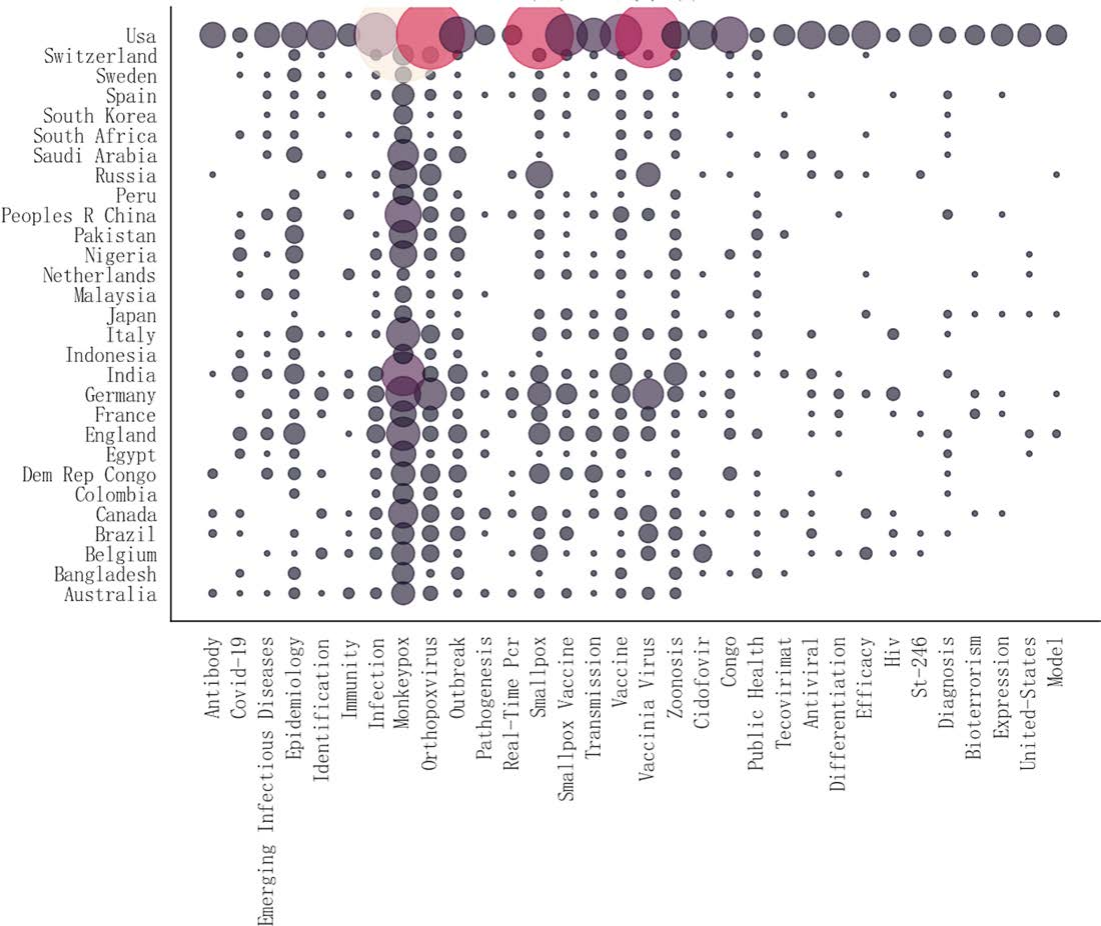
Focus item in countries.

Regarding terminologies, epidemiology, emerging infectious diseases, outbreak, orthopoxvirus, smallpox, smallpox vaccine, infection, transmission, and zoonosis were the common topics of concern in most countries. At the country level, the United States focused on various topics, as shown in Figure 3, with particular attention to Mpox, Mpox-related orthopoxvirus (e.g., smallpox and vaccinia virus), and drugs used to treat Mpox (e.g., ST-246 and Cidofovir). In Russia and Germany, the main topic of interest was Mpox-related orthopoxvirus. The United States, Germany, France, the United Kingdom, and Canada were concerned about several topics, whereas most of the other countries had research gaps in topics such as models, bioterrorism, Mpox-related HIV, efficiency, antiviral, antibodies, and drugs for Mpox, indicating that there is still much room for expansion in research in those countries. As shown in Figure 3, the U.S., Germany, France, the United Kingdom, and Canada demonstrated relatively diversified research focuses, while other countries exhibited research gaps in certain fields. This suggests these nations could strengthen collaborations with countries possessing expertise in relevant areas to expand their research directions.

### 3.4. Institutional analysis of the documents on Mpox

Table 2 shows the top 15 high-producing institutes, ranked by the number of publications. All the top 4 institutes were from the United States, with the CDC leading the pack by a wide margin. The next top 10 institutes were from the United States included the National Institutes of Health, University of California System, National Institute of Allergy Infectious Diseases, Harvard University, Emory University, and Johns Hopkins University. In terms of agency functions, the CDC provides guidance on disease prevention and control and responds to public health emergencies. The National Institute of Allergy Infectious Diseases mainly conducts basic and applied research on infectious, immune, and allergic diseases. All these institutions have a deep research background in the prevention and treatment of infectious diseases. The WHO ranked eighth, with a high *H* index of 37, following the CDC and Nation Institutes of Health. Egyptian Knowledge Bank in Egypt ranked fifth, but has low cited frequency and H index. Institute Nacional De Recherche Biomedical in the France, and University of Texas System in the United States ranked 11th simultaneously. King Saud University in Saudi Arabia ranked thirteenth, University College London in the United Kingdom ranked 14th, University Paris City in France ranked fifteenth but with a relatively small H index.

**Table 2:** Table of high yielding institutions on Mpox study.

Number	Institution	Number of publications	Times cited	Average per item times cited	*H* index
1	Centers for Disease Control Prevention	289	14,544	50.33	66
2	Nation Institutes of Health	118	5969	50.58	41
3	University of California System	105	4182	39.83	30
4	National Institute of Allergy Infectious Diseases	87	3925	45.11	33
5	Egyptian Knowledge Bank	84	662	7.88	14
6	University of London	79	5313	67.25	25
7	Harvard University	78	1705	21.86	24
8	World Health Organization	68	6238	91.74	37
9	Emory University	55	1823	33.15	21
10	Johns Hopkins University	54	1319	24.43	16
11	National Institute of Biomedical Research	51	2255	44.22	23
11	University of Texas System	51	1857	36.41	17
13	King Saud University	48	278	5.79	10
14	University College London	47	3100	65.96	20
15	University Paris City	46	1216	26.43	16

Clustering of institutions according to the intensity of interinstitutional collaboration resulted in 4 clusters centered on the CDC, Saint Louis University, Harvard Medical School, and Emory University in the United States (Appendix Fig. S2, Supplemental Digital Content, https://links.lww.com/MD/P409). The CDC cluster was dominated by institutions at the national level, such as National Institute of Allergy and Infectious Diseases, National Institutes of Health, Food and Drug Administration in the United States, Ministry of Health of various countries, State Research Center of Virology and Biotechnology Vector in Russia. CDC also worked with universities in other countries. For example, the CDC, in collaboration with Kinshasa School, conducted a prospective cohort study in 2018 to examine the effectiveness of a novel smallpox vaccine against Mpox infection. Universities are the dominant institution type in Saint Louis University – centered cluster, some of which are University of Pennsylvania, University College London, Chinese Academy of Sciences, University of Oxford, Stanford University, University of Florida, Peking University. Harvard Medical School – centered cluster include institutes such as Lebanese American University, Massachusetts General Hospital. Emory University – centered cluster include institutes such as King Saud University, Columbia University, Johns Hopkins University.

**Figure 4.**
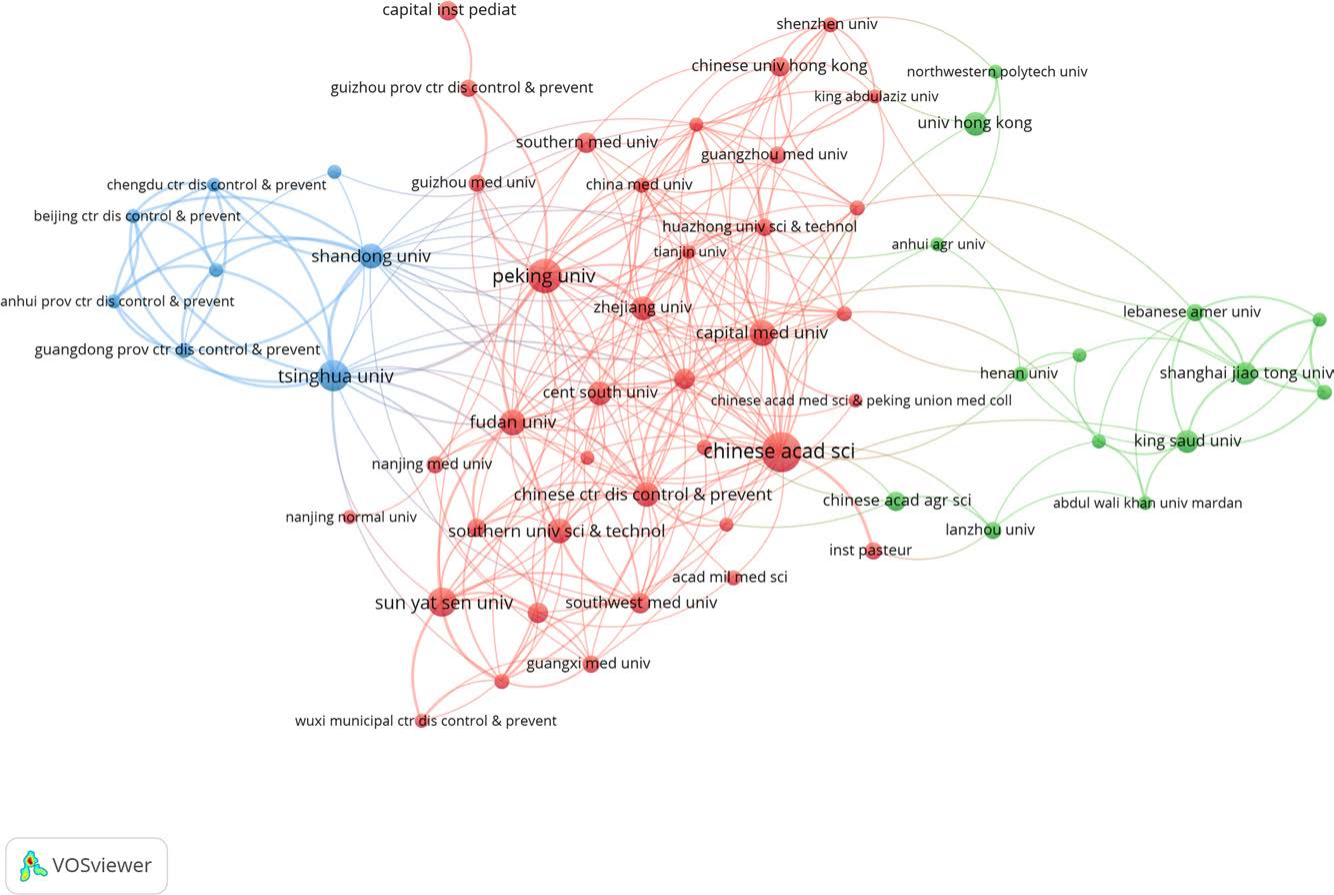
Network map of institutes cooperation in China.

As for Institutes cooperation in China, it can be divided into 3 cluster (Fig. 4). Tsinghua University and Shandong University mostly cooperate with provincial centers for disease control and prevention, such as Chengdu center disease control and prevention, Anhui center disease control and prevention, Guangdong center disease control and prevention, Beijing center disease control and prevention. The cluster represented by the Chinese Academy of Sciences and Peking University is dominated by universities, such as Central South University, Capital Medical University, Fudan University, Zhejiang University, and Sun Yat Sen University. The third cluster is centered on Shanghai Jiao Tong University and there are more international universities in this cluster, such as Lebanese American University, King Saud University. As shown in Figure 4, in China, in addition to interuniversity collaborations, academic institutions tend to cooperate with provincial CDCs. This pattern may be attributed to CDCs’ access to more comprehensive Mpox case data, while domestic universities also maintain research partnerships with international academic institutions.

### 3.5. Journal analysis of the documents on Mpox

The analysis of journals published on Mpox provides an understanding of the main Mpox publications groups and the preferences of different journals, which can help authors who are new to the field review corresponding papers and make submission decisions. The top 5 journals in terms of the number of published papers and citation frequency are shown in Table 3 with *Viruses-Basel* having the highest number of publications (53) and *Journal of Virology* having 2277 citations, indicating that the quality of its articles is widely recognized.

**Table 3:** The number of publications and citations in Top 5 Journals.

Journal	Number of publications	Times cited	Average per item	*H*-index
Journal of Virology	105	1017	9.69	16
Viruses-Basel	90	2043	22.7	22
Vaccines	69	662	9.59	16
Emerging Infectious Diseases	66	3465	52.5	29
Vaccine	62	1854	29.9	24

### 3.6. Authorship analysis of the documents on Mpox

Authors are the main participants in research work. In this study, we identified high-yield and high-producing teams and tried to understand the author clusters of specific topics as well as the cooperative relationship between these authors, which is conducive to further exploring the advantageous research directions of different research teams.

#### 3.6.1. Analysis of major authors

Table 4 presents the top 10 authors based on publication numbers, along with the average citations of their works. The leading author was Damon IK, who works at the CDC and is a part-time faculty member at Emory University, setting the stage for collaboration between the 2 institutions. The authors’ research focused on a variety of topics. For example, Damon IK’s research focuses mainly on orthopoxvirus such as smallpox, antiviral drugs, smallpox vaccine, and Ebola virus. Carroll DS pays special attention to smallpox, vaccinia virus, animal models, and drugs against smallpox. Li Y’s main research interest is in the epidemiology of Mpox. All the top 7 authors are from the CDC. Hruby DE, ranked eighth, is from SIGA and has coauthored an article with Damon IK on therapeutic drugs against Mpox. The ninth-ranked author was Jahrling PB from the National Institute of Allergy Infectious Diseases. The 10th-ranked author is from the Robert Koch Institute. Overall, the high-yield authors are affiliated with the high-producing institutes.

**Table 4:** The average citations of the top 10 authors.

Ranking	Author	Number of papers	Average citations
1	Damon IK	88	86.95
2	Reynolds MG	65	72.46
3	Carroll DS	39	29.95
4	Olson VA	35	30.06
5	Karem KL	33	32.36
6	Mccollum AM	30	37.07
7	Li Y	25	34.52
8	Hruby DE	22	37.14
9	Jahrling PB	19	24.74
10	Nitsche A	18	17.94

#### 3.6.2. Analysis of author collaboration networks

**Figure 5.**
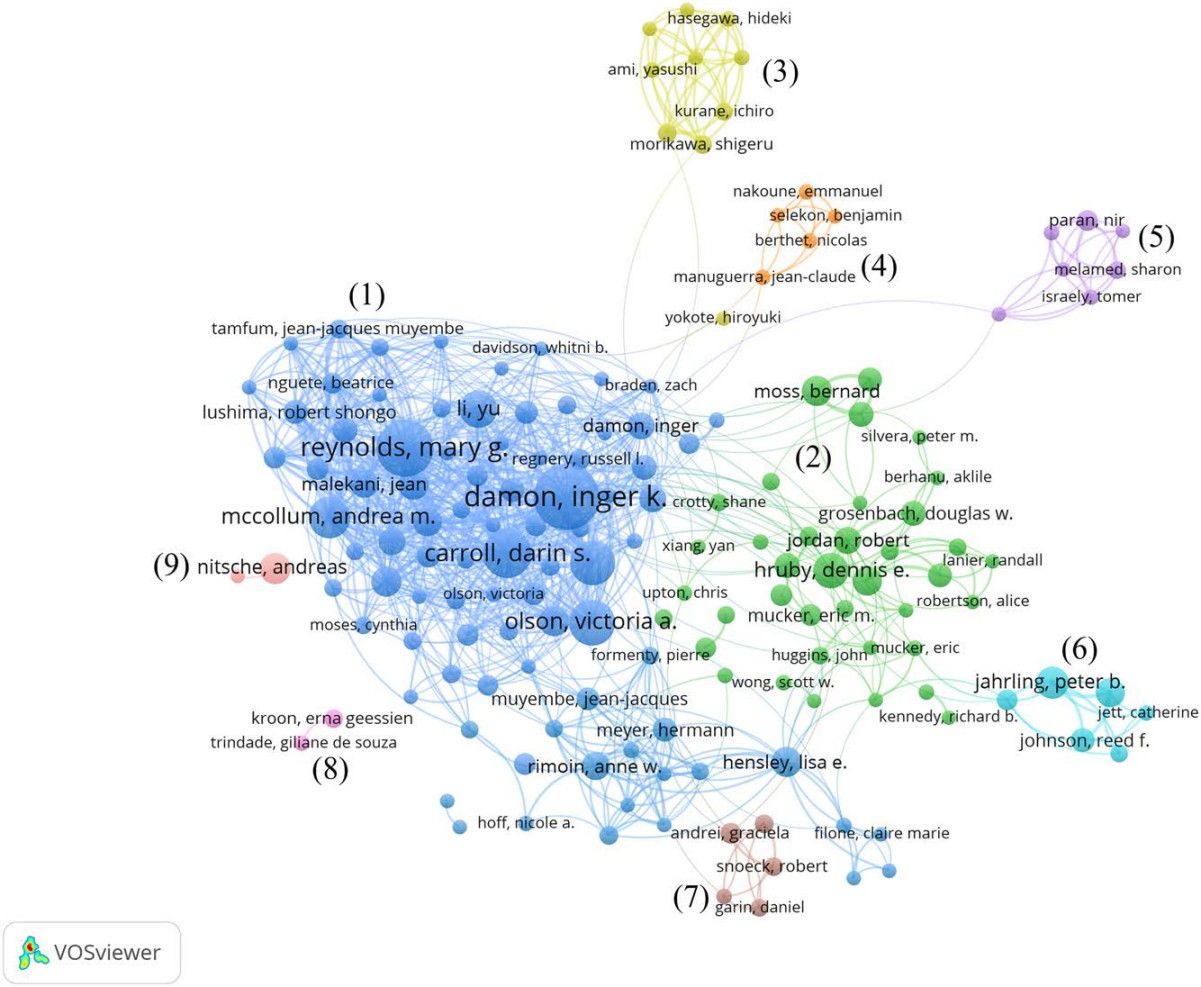
Author cooperation network.

The authors were divided into 9 clusters based on the collaborative network analysis (Fig. 5). The largest author cluster included Damon IK, Reynolds MG, Carroll DS, and Olson VA. All these were from the CDC, and this cluster has significant strength and influence in Mpox research. The second largest author cluster included Hruby DE and Jordan R, and they are both from SIGA. The other collaborating clusters are relatively independent. These clusters include a cluster from the National Institute of Infectious Diseases in Japan, a cluster from Institut Pasteur and Institut Pasteur of Shanghai Chinese Academy of Sciences, a cluster from Israel Institute for Biological Research, a cluster from the National Institute of Allergy Infectious Diseases, a cluster from Katholieke Universiteit Leuven, a cluster from Universidade Federal de Minas Gerais, and a cluster from Robert Koch Institute. This suggests that the U.S. CDC holds significant influence in Mpox research, with researchers primarily forming collaborative teams through institutional affiliations.

### 3.7. Analysis of research directions of documents on Mpox

#### 3.7.1. Keywords analysis of the document on Mpox

**Figure 6.**
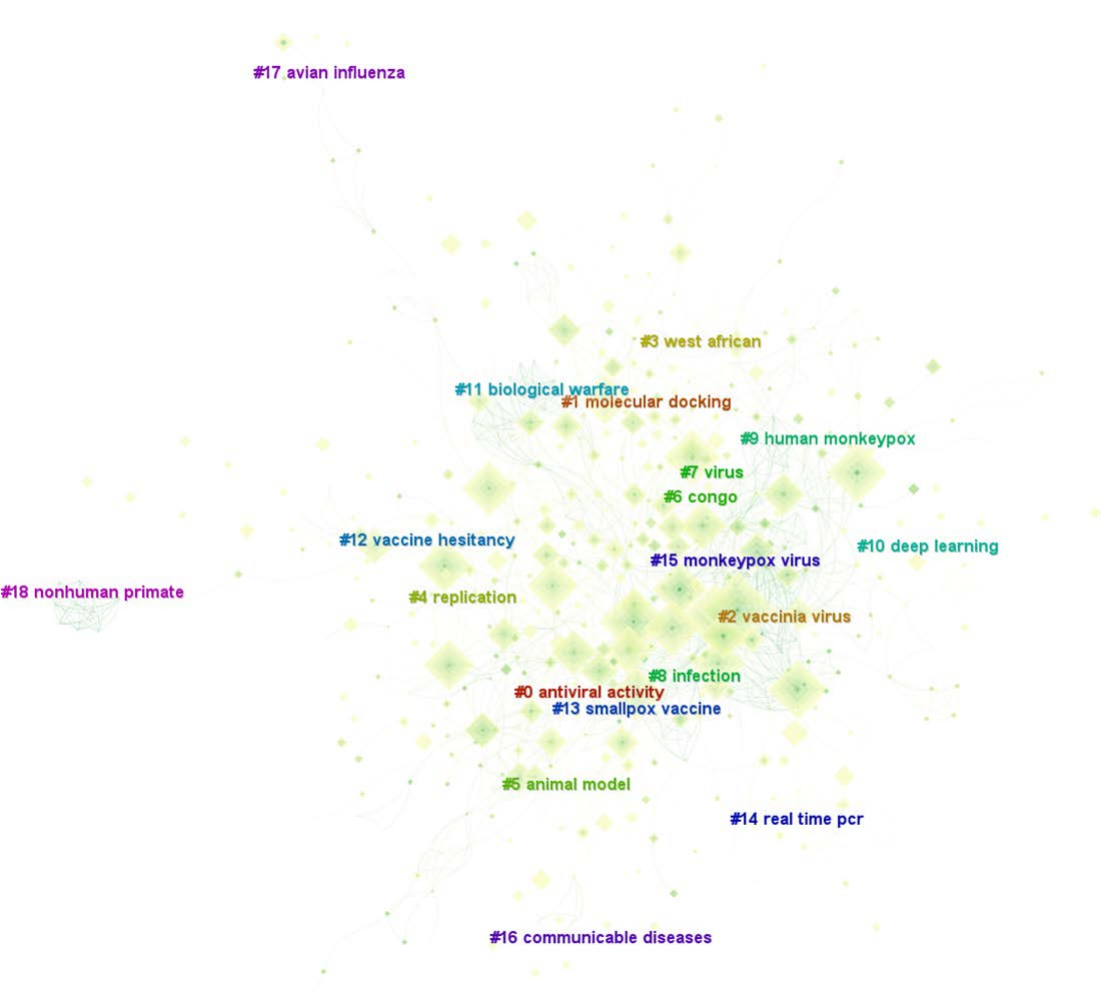
Keyword clustering of the documents on Mpox.

The analysis of disciplinary structure and knowledge foundations, as visualized in Figure 6, was conducted through keyword clustering analysis. This process identified 19 distinct thematic clusters that, upon systematic literature review, were consolidated into 5 primary research domains (Table 5).

**Table 5:** Five research directions and 19 thematic clusters.

Research directions	Thematic cluster
Epidemiology and public health regulation	#3, #6, #8, #9, #15, #17
Genetic evolution and mechanisms of transmission	#1, #16
Pathogenesis and host immunity	#5, #18, #4
Drugs against Mpox research and efficacy of related vaccines	#0, #2, #7, #12, #13
Research on early diagnosis and its new method	#10, #14
Biological warfare potential of Mpox	#11

#### 3.7.2. Co-citation analysis of the documents on Mpox

Further, we used direct citation relationships to develop a citation network, focusing on highly cited and high betweenness-centrality papers in each category and manually interpreted the research content as well as the core ideas.

##### 3.7.2.1. Epidemiology and public health regulation

An infectious disease caused by the Mpox virus has been an epidemic in more than a dozen African countries. The Mpox outbreak has received widespread attention since early May 2022, as Mpox viral infections have been reported in many non-endemic countries. In recent Mpox epidemics, the main affected groups are men who have sex with men (MSM).^[21]^ According to relevant national reports, the proportion of patients with HIV among known cases of Mpox infection in the MSM population is more than 20%.^[22,23]^

##### 3.7.2.2. Genetic evolution and mechanisms of transmission

Mpox viruses are divided into 2 clades based on geographic factors and genetic and phenotypic differences. They are genetically conserved, with approximately 95% homology between the 2 clades. In general, Clade II has lower attenuation and higher transmission capacity than that observed in Clade I.^[24]^ A related study analyzed the genome of Mpox virus associated with the 2022 outbreak and found that the majority of the mutations in the genome were point mutations, that is, single nucleotide substitutions.^[25]^ This genomic change may be related to the adaptive advantage of orthopoxvirus evolution.

The 2 elements of disease emergence are the introduction of pathogens into the population and the disease transmission and maintenance in the population. Disease transmission is mainly influenced by evolutionary and ecological factors. Evolutionary factors mainly refer to changes in the virulence of the pathogen during the process of growth adaption in humans and the subsequent spread from person to person. Ecological factors refer to human behavior, changes in host density, etc. Among them, ecology plays an important role in the emergence of the disease, and ecological changes may lead to an increased possibility of Mpox infections in humans.^[26]^

##### 3.7.2.3. Pathogenesis and host immunity

Studying the host species, geographical distribution of hosts, and infection route of Mpox virus can lay the foundation for further understanding of the pathogenic mechanism of Mpox virus.^[27]^ The natural host of Mpox has not been identified; however, rodents are the most likely hosts.

In the course of human infection, Mpox typically has an incubation period of 6 to 13 days or 5 to 21 days. The infection is usually characterized by fever, swollen lymph nodes, back pain, muscle pain, and weakness. The rash usually begins within 1 to 3 days after a fever and tends to be concentrated on the face, extremities, palm, and soles of the feet, and the oral mucous membranes, genitalia, conjunctiva, and corneas may be affected.^[28]^ Symptoms typically last 2 to 4 weeks, and the duration of illness is related to the degree of viral exposure, health status of patients, and nature of complications. In recent years, the mortality rate of Mpox has ranged from 3% to 6% and is higher in young children.^[29]^

##### 3.7.2.4. Drugs against Mpox research and efficacy of related vaccines

Drugs currently available for Mpox treatment include Tecovirimat, Brincidofovir, and Cidofovir, and approved vaccines include JYNNEOS and ACAM2000.^[30]^ Cidofovir has selective activity against broad-spectrum DNA viruses, such as herpes simplex virus and vaccinia virus.^[31]^ Further development of Cidofovir has led to the development of Brincidofovir (also known as CMX001), which is more potent but cytotoxic and has been approved by the United States Food and Drug Administration for the treatment of smallpox.^[32]^ However, its efficacy in the treatment of Mpox has not been validated, but in efficacy evaluations in animal models, Brincidofovir showed a protective effect.^[33]^ In vitro studies have shown that Tecovirimat (also known as ST-246) can be used as an antiviral therapeutic compound as it blocks the production and release of the virus.^[34]^ Based on data from animal and human studies, the European Medicines Agency approved Tecovirimat in 2022, an antiviral drug for smallpox developed by SIGA for the treatment of Mpox.^[35]^

##### 3.7.2.5. Research on early diagnosis and its new method

Early diagnosis of Mpox is very important as it is a transmitted disease. Currently, Mpox virus can be identified by measuring antibodies and antigens, assessing DNA, or characterization. Polymerase chain reaction (PCR) is the preferred laboratory test due to its accuracy and sensitivity. GeneXpert is a system that can combine sample preparation with PCR amplification and detection, which can minimize contamination and shorten detection time.^[36]^ In some locations where PCR is not available, nucleic acid amplification test, a viral diagnostic test, can be used for Mpox detection and has shown high sensitivity and specificity.^[36]^

It is worth mentioning that with the development of artificial intelligence, this test can also play a supporting role in disease diagnosis. For example, Mpox infection image datasets are used for identification and classification.^[37,38]^

##### 3.7.2.6. The biological warfare potential of Mpox

Since the eradication of smallpox in the 1970s, Mpox, which has been the most prominent orthopoxvirus responsible for human epidemics, has been considered a potential biological weapon.^[39]^ Studies have shown that naturally occurring Mpox may not pose a serious bioterrorism threat due to its low fatality rate and limited ability to spread.^[40]^ However, the virus can be genetically manipulated to gain greater virulence and transmission capacity, so it is necessary to be wary of related manipulations based on genetic engineering.^[39]^

#### 3.7.3. Analysis of research hotspots

By drawing the keywords outburst map (Appendix Fig. S3, Supplemental Digital Content, https://links.lww.com/MD/P409), we found that from 2000 to 2008, research on the pathogenic biology of Mpox was a hot research direction. From 2008 to 2019, research on the development of drugs against Mpox and animal models was the hot research direction. From 2019 to the present, research on the epidemiology of and public health issues on Mpox have been the hot research direction. With the increase in confirmed Mpox cases among MSM, clinical data on Mpox combined with HIV infection have increased, and related studies, including changes in immunological parameters and drug administration methods, have gradually become hot research directions.^[41,42]^ Overall, each stage has a more dominant research content. Consistent with the disciplinary distribution of the publications, the research tilted from basic research to application-oriented research.

## 4. Discussion

In this study, 3401 papers retrieved from the WoS database were visually analyzed by VOSviewer, CiteSpace, and data-information-knowledge-wisdom bibliometric software. Regarding the total number of publications and citation frequency, the United States ranked first, with the CDC being the key institution for research on Mpox. Regarding authorship, most of the authors with high publications and high citations were from the CDC. Regarding country cooperation, the United States is at the center of cooperation. In terms of institute collaboration, the United States has more diverse partnerships such as national-level institutions and universities and companies, while Chinese institutions mostly cooperate with nation-level institutes in other countries rather than with companies and universities.

In terms of research topics, epidemiology and public health regulation, genetic evolution and mechanisms of transmission, and pathogenesis and host immunity are relatively the traditional research direction. Studies on the transmission mechanism combined with ecology and artificial intelligence-based methods for the diagnosis of Mpox are emerging research directions.

## 5. Conclusion

The current global landscape of Mpox prevention and control remains highly challenging, posing significant threats to international health security. Mpox is a mandatory reportable disease in the Democratic Republic of Congo; however, in most affected countries, especially in parts of the countries with active Mpox outbreaks, health services are under-resourced. Remote rural areas lack health care isolation areas and personal protective equipment, and many areas are active sites of conflict and civil unrest; therefore, Mpox is not systematically included in their integrated disease detection and response systems. The absence of a good disease surveillance system may hamper early detection and response, which poses significant challenges to the management of Mpox outbreaks worldwide.

Recent years have witnessed an increasingly pressing trajectory of epidemic development coupled with persistent surveillance dilemmas. In recent years, the geographic scope and frequency of Mpox outbreaks have increased, posing new challenges to public health regulation in various countries. Many factors, such as seasonal nomadism, refugee movement, and cross-border economic exchange, affect Mpox surveillance. In some severely affected countries, the lack of standardized case definitions and inadequate training in health care has prevented them from conducting systematic Mpox surveillance and collecting as well as reporting relevant data. Therefore, it is important to provide the necessary support for prevention, case detection, and laboratory development in countries where Mpox is endemic and regulatory systems are weak. There is a need to adhere to the “One Health” mentality, mechanisms for cross-border communication, and information sharing.

Regarding key priorities for containment and research, more attention should be paid to the MSM population, and the dissemination of basic knowledge of Mpox disease among the MSM population should be strengthened. On the other hand, the variation of the Mpox genome and assumption that the variation is an evolution should be monitored closely in scientific research. This will enable researchers to focus on what caused this evolution. In terms of transmission, relevant studies should emphasize ecological factors in the virus transmission model. In most studies, researchers tend to communicate that stakeholders should not be worried about Mpox being used as a biological weapon, but it is necessary to be alert to the threat posed by its modification caused by biotechnology.

From the perspective of technological empowerment, as an emerging and rapidly developing field, artificial intelligence has made significant progress in the detection, screening, diagnosis, and classification of Mpox, as well as the characterization of Mpox viral genomes and assessment of Mpox drug utility. Using artificial intelligence to identify disease clusters, monitor cases, and predict outbreaks is a new trend in disease research. New technologies and methods in Mpox research should be strengthened, with emphasis on disease surveillance, regional approaches to enhance disease prevention and control and mapping of ecological risk niches.

This study employs bibliometrics to conduct a comprehensive analysis of Mpox-related research literature. However, several limitations should be acknowledged regarding the research scope. Although the WoS database represents scientific achievements in global research, our analysis was exclusively confined to WoS-indexed publications. Notably, our study emitted other major databases like Scopus and native-language databases from non-English speaking countries. Future studies should expand the literature scope to enable more comprehensive bibliometric analyses in the Mpox field. The main obstacles that may impede progress include interpretation and analysis of keyword clustering as well as evolutionary trend analyses.

## Author contributions

**Conceptualization:** Qunjiao Yan.

**Formal analysis:** Qunjiao Yan.

**Methodology:** Qunjiao Yan.

**Software:** Qunjiao Yan.

**Visualization:** Qunjiao Yan.

**Writing – original draft:** Qunjiao Yan.

**Writing – review & editing:** Lei Wang.

## Supplementary Material


