# Supplementary material for: A bibliometric analysis of Mpox research based on Web of Science platform
Source: Medicine (Baltimore). 2025 Jul 11;104(28):e43329. doi: 10.1097/MD.0000000000043329 (PMC12263044; doi:10.1097/MD.0000000000043329)

Appendix Figure 1 Discipline Ranking in 2022


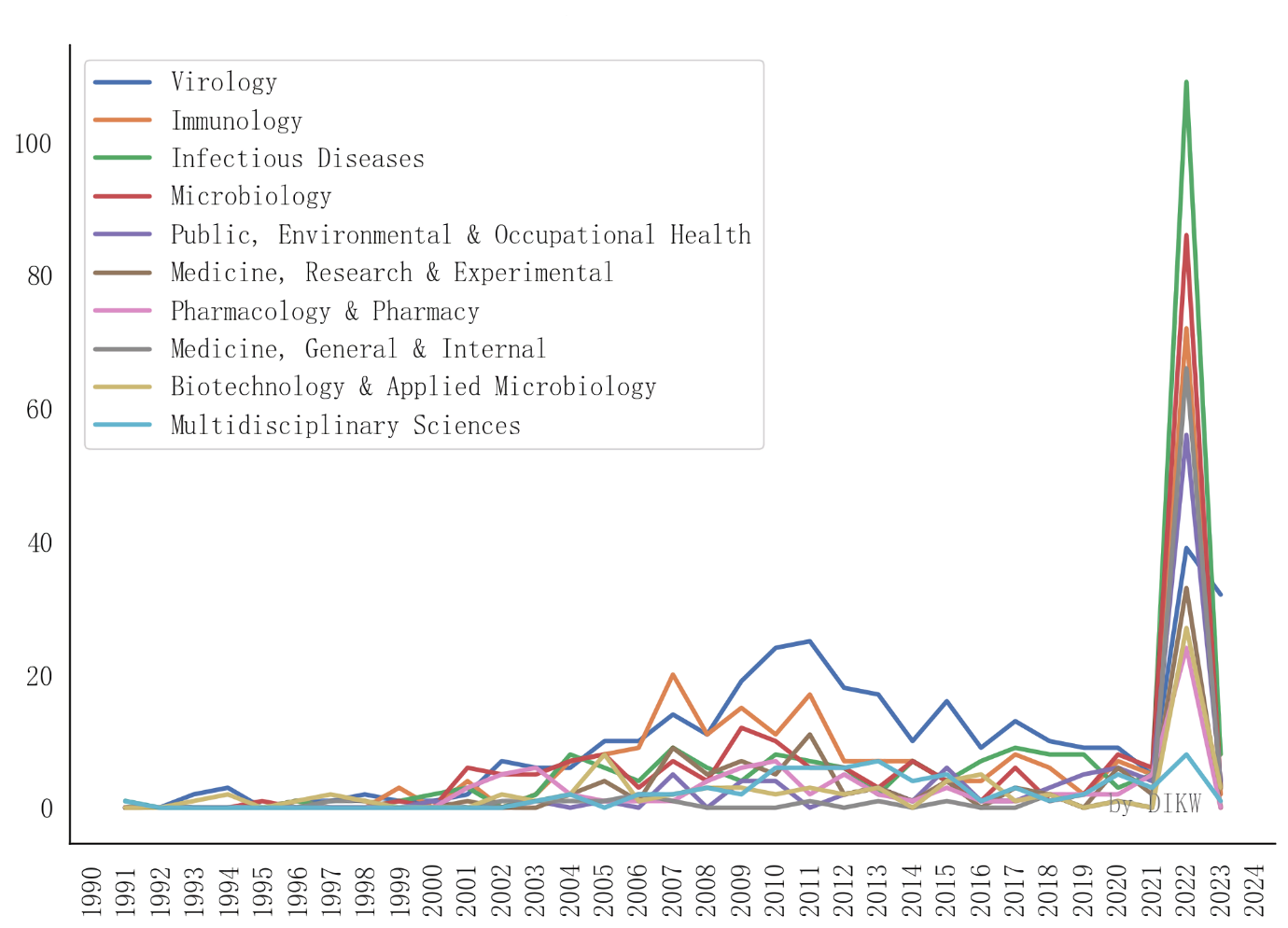


Appendix Figure 2 Institution Cooperation Network


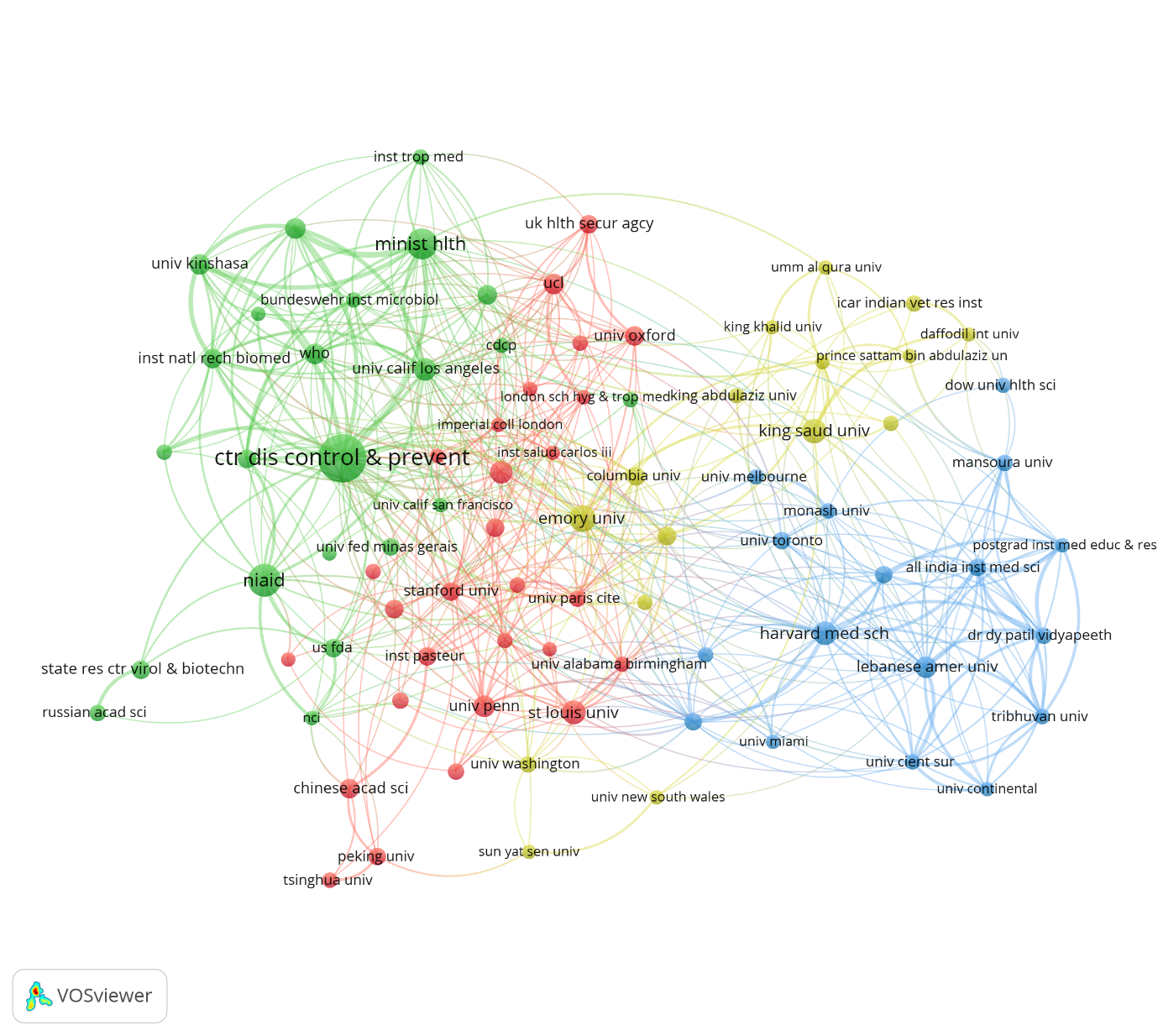


Appendix Figure 3 Keywords Outburst Map


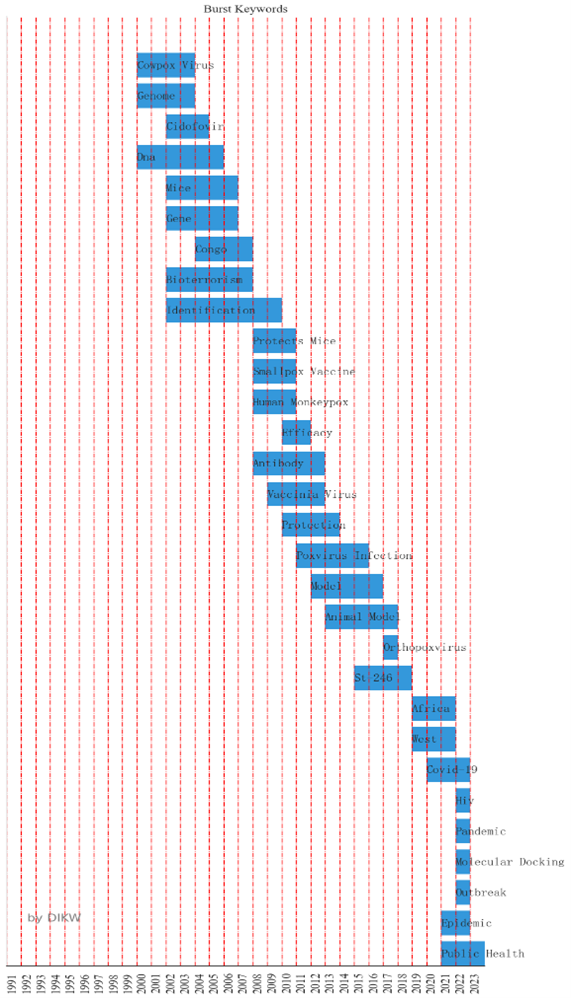

Supplement: Supplementary file 1 [file medi-104-e43329-s001.docx]
